# Median and Ulnar Neuropathy Assessment in Parkinson's Disease regarding Symptom Severity and Asymmetry

**DOI:** 10.1155/2016/4958068

**Published:** 2016-10-24

**Authors:** Nilgul Yardimci, Ozlem Cemeroglu, Eda Ozturk, Gülsüm Gürlü, Esra Şahin, Saliha Bozkurt, Tugba Cengiz, Gulderen Karali, Hasim Cakirbay, Atilla İlhan

**Affiliations:** ^1^Neurology Department, Minasera Aldan Hospital, Ahmet Taner Kislali Mah. 2741, Street No. 2 Cayyolu, Ankara, Turkey; ^2^Physical Medicine and Rehabilitation Department, Medical Park Ankara Hospital, Kentkoop Mah., Kentkoop Parkici Yolu, Yenimahalle, Ankara, Turkey; ^3^Biostatistics Department, Medicine Faculty, Hacettepe University, Hacettepe Mah., 06230 Ankara, Turkey; ^4^Physical Medicine and Rehabilitation Department, Medicine Faculty, Turgut Ozal University, Alparslan Turkes Cad. No. 57, Emek, 06510 Ankara, Turkey; ^5^Neurology Department, Medicine Faculty, Turgut Ozal University, Alparslan Turkes Cad. No. 57, Emek, 06510 Ankara, Turkey; ^6^Neurology Department, Medicine Faculty, Gazi University, Emniyet, Yenimahalle, 06560 Ankara, Turkey

## Abstract

*Background*. While increasing evidence suggests comorbidity of peripheral neuropathy (PNP) and Parkinson's disease (PD), the pathogenesis of PNP in PD is still a debate. The aim of this article is to search the core PD symptoms such as rigidity and tremor as contributing factors to mononeuropathy development while emphasizing each individual patient's asymmetric symptom severity.* Methods*. We studied 62 wrists and 62 elbows of 31 patients (mean age 66.48 ± 10.67) and 64 wrists and 64 elbows of 32 age-gender matched healthy controls (mean age 62.03 ± 10.40, *p* = 0.145). The Hoehn and Yahr disability scale and Unified Parkinson's Disease Rated Scale were used to determine the severity of the disease.* Results*. According to electrodiagnostic criteria, we confirmed median neuropathy in 16.12% (bilateral in two-thirds of the patients) and ulnar neuropathy in 3.22% of the PD group. While mean age (*p* = 0.003), age at PD onset (*p* = 0.019), and H&Y scores (*p* = 0.016) were significant, tremor and rigidity scores were not. The comparison of the mean indices of electrophysiologic parameters indicated subclinical median and ulnar nerve demyelination both at the wrist and at the elbow in the patient groups where a longer disease duration and mild tremor and rigidity scores are prominent, remarkably.* Conclusion*. A disease related peripheral neurodegeneration beyond symptom severity occurs in PD.

## 1. Introduction

Parkinson's disease (PD) is the second most common neurodegenerative disorder, characterised by tremor, rigidity, bradykinesia, and postural instability associated with degeneration of dopaminergic neurons in the substantia nigra pars compacta and the presence of eosinophilic intracytoplasmic inclusions [1].

Identifying comorbid conditions in addition to the core PD symptoms may increase satisfaction from traditional therapies by maximizing the health status of the patient. In accordance with this, the possible relationship between PD and peripheral neuropathy (PN) has begun receiving attention. Although the pathogenic mechanism remains unclear, some studies suggest PN as a neurodegenerative process involving both central and peripheral nervous systems [2]. It is still a debate whether PN in PD should be accepted as an acquired neuropathy due to long-term levodopa intake or as an intrinsic neuropathy developed by neurodegeneration involving the peripheral nervous system in PD pathophysiology.

Although by advancing therapeutic options more successful symptom control has been provided in PD patients today, rigidity and tremor still remain the challenging motor symptoms of the disease. Besides being one of the cardinal symptoms of PD, the characteristic rest tremor seen as “pill-rolling” action of the hands has been also accused of being the trigger of the carpal tunnel syndrome because of causing repetitive trauma to the median nerve [3, 4]. Striatal hand and foot deformities occur in varying degrees associated with the disease severity and may cause functional disability also. The pathogenesis of striatal deformities involves decreased striatal dopamine, dystonia, and parkinsonian rigidity [5]. The classical position of the elbow in accordance with the striatal deformities of the PD which is superimposed with tremor has not been searched as the potential risk for ulnar neuropathy before [6].

In this study, we aimed to search if the severity of clinical symptoms such as tremor and rigidity affects neuronal transmission and contributes to entrapment neuropathy evolution in PD. In regard to this, we also investigated if each patient's initial and latter affected extremities which demonstrate varying symptom severity during the progressive disease course differ electrophysiologically. These hypotheses are being searched for the first time in the literature and aim to call attention to peripheral neuropathy while planning therapeutic options in each individual PD patient.

## 2. Patients and Methods

### 2.1. Parkinson's Disease Group

Patients were recruited from the Neurology Outpatient Unit in the Department of Neurology, Turgut Ozal University Faculty of Medicine. Consecutive patients who attended the clinic were examined by a neurologist and those who fulfilled the study criteria were entered in the study. 40 patients were enrolled in the study. Among these patients, 1 was excluded from the study because of previous left elbow fracture, 3 had diabetes mellitus, and 5 had polyneuropathy. Totally, 62 elbows and 62 wrists of 31 patients with PD diagnosed by means of the United Kingdom Parkinson's Disease Society Brain Bank Clinical Diagnosis Criteria [7] [18 women (58.1%) and 13 men (41.9%)] with an age range of 45–83 years (mean age, 66.48 ± 10.67 years) were included in the study. Exclusion criteria included coexistence of any systemic disease known to cause median or ulnar neuropathy such as diabetes mellitus, chronic renal failure, hypothyroidism, rheumatoid arthritis, cervical radiculopathy, and trauma. All patients were examined in the “on” condition. The Hoehn and Yahr (H&Y) disability scale [8] and Unified Parkinson's Disease Rated Scale (UPDRS) [9] were used to assess the severity of the disease. The patients were not symptomatic regarding pain or numbness suggesting nerve entrapment in the territory of a root or nerve.

Firstly, the patients were examined for existence of any median or ulnar neuropathy according to the electrophysiologically diagnostic criteria based on control data performed in our laboratory.

In elderly subjects such as our study population, subclinical neuropathy is common and might increase the frequency of mononeuropathy. We compared the means of electrophysiological indices of the patients and the controls for any statistically significant difference also.

As known, PD symptoms begin unilateral initially and in the following years the initial affected extremities demonstrate more severe symptoms when compared with the latter affected ones. Keeping this rule in mind, we compared the initial and latter affected sides of each patient electrophysiologically in regard to tremor and rigidity severity.

Later, each patient was categorized as mild or severe according to the H&Y scores, tremor and rigidity severity, and disease duration. These groups electrophysiologic parameters were compared statistically.

### 2.2. Comparison Group

64 elbows and 64 wrists of 32 healthy controls [22 women (68.8%) and 10 men (31.3%)] with an age range of 46–87 years (mean age, 62.03 ± 10.40, *p* = 0.145) who had no disease known to cause median or ulnar neuropathy, no history of frequent, repetitive use of the hand, wrist, or elbow, and no trauma or any other condition affecting peripheral neural transmission and whose neurologic examination was normal were included in the study. The subjects were volunteers from the community matched by age and sex with the PD patients. Any subject with electrophysiological pathology was excluded from the study.

Informed consent was obtained from all individual participants included in the study and the study was approved by Turgut Ozal University Medicine Faculty Ethical Committee.

### 2.3. Electrophysiological Evaluation

Sensory nerve conduction velocity (SNCV) and motor nerve conduction velocity (MNCV) were measured with a Medelec Synergy Electromyography instrument (Oxford Instruments Medical, Inc., UK) in an air-conditioned room at 23°C to 25°C. Limb temperature was maintained above 32°C using a warm compress if needed during the nerve conduction studies. Median and ulnar nerve conduction studies were performed bilaterally in all patients and control groups using the standard techniques of supramaximal percutaneous stimulation [10].

The EMG criteria for diagnosis of median neuropathy include prolonged distal motor latencies of the median nerve greater than 4.2 m/s, a decrease in sensory conduction velocity below 41 m/s for digit-wrist segment, 5 mV for amplitude of muscle action potential, and 10 mV for amplitude of sensory nerve action potential [11].

The EMG criteria for diagnosis of ulnar neuropathy include prolonged motor nerve conduction velocity (NCV) from above elbow (AE) to below elbow (BE) of less than 40 m/s and an AE to BE segment greater than 10 m/s slower than BE to wrist (W) segment which suggests conduction block or temporal dispersion indicative of focal demyelination.

### 2.4. Statistical Analysis

For continuous variables, results were presented as mean ± standard deviation or median (minimum, maximum). Categorical variables were presented by frequency and percentage. Comparisons between two categorical variables were performed using the chi-square analysis. Kruskal-Wallis test or one-way ANOVA (analysis of variance) were used for comparison of more than two independent samples followed by the Bonferroni-adjusted Mann–Whitney *U* test or Tukey test when appropriate. All analyses were performed using IBM SPSS Statistics 21 (IBM, Armonk, NY USA). All tests were two-sided and a *p* value of less than 0.05 was considered to be statistically significant.

## 3. Results

All patients were receiving levodopa and/or dopaminergic agonist and were examined during “on” condition. The clinical characteristics of the study population are summarized in Table 1.

According to diagnostic electrophysiologic criteria, 10 wrists (16.12%) in 6 patients were diagnosed as having definite median neuropathy and 2 elbows (3.22%) in 2 patients were diagnosed as having ulnar neuropathy. The electrophysiologic parameters of the healthy control group were in normal limits. The clinical characteristics of the patients with median neuropathy are listed in Table 2. Median neuropathy was diagnosed as bilateral in 4 patients and unilateral in 2 patients. Among the patients with median neuropathy, mean age (*p* = 0.003), age at Parkinson disease onset (*p* = 0.019), UPDRS III motor score (*p* = 0.005), Hoehn and Yahr stage (*p* = 0.023), and Parkinson disease questionnaire scores (*p* = 0.016) were statistically significantly higher. Tremor (*p* = 0.390) and rigidity (*p* = 0.059) scores which we were particularly interested in were not significantly different.

As the ulnar neuropathy in this study was observed in 2 patients representing 3.2% of the study population, we did not mention the patient details in Table 2 but preferred to explain them as follows: the first patient was a 75-year-old male with a 2-year duration of disease scored as 2 according to H&Y stage with mild tremor and rigidity scores and the second patient was a 64-year-old female with a 10-year duration of disease scored as 4 according to H&Y stage with severe tremor and rigidity.

The comparison of the means of electrophysiological indices on the left and right sides between controls and the patients is shown in Table 3. While right and left median nerve sensorial velocities at the wrist were decreased, right and left median nerve motor latencies were prolonged statistically significantly indicating demyelination. There was a significant decrease in the right median nerve motor amplitude whereas the decrease in the left median nerve motor amplitude did not reach statistically significant levels. It might be suggested that there was a possible median nerve axonal deterioration also. While there was a significant decrease in the left ulnar nerve sensorial velocity at the wrist, the decrease in the right ulnar nerve did not reach significant level. The prolongation of the right and left ulnar nerves' motor latencies were significant. These findings suggested ulnar nerve demyelination at the wrist when compared with the age-gender matched controls. The reduction in the left ulnar nerve motor velocity at the elbow level was significantly decreased while the reduction in the right was not significant. These results suggested a tendency to ulnar nerve entrapment at the elbow in the patient group.

In concordance with our hypothesis that more severe tremor and rigidity might worsen neuronal transmission, we compared each patient's median and ulnar nerve electrophysiologic parameters between the initial and latter affected extremities (Table 4). All parameters regarding ulnar and median nerves were studied and mean indices were compared with each other. No significant difference was found but if we could have learned the time span between the first and second affected sides of the body for each patient accurately and categorized it as years, the results might have been more objective.

Later, each patient was categorized as mild or severe according to the mentioned criteria; Mild H&Y group (stage I-II) consisted of 14 patients (45.16%) with an age range of 45–76 years (mean age 63.15 ± 10.51) and the severe H&Y group (stage III-IV) consisted of 17 patients (54.83%) with an age range of 47–82 years (mean age 69.63 ± 9.75, *p* = 0.084). In our study, mean of the disease duration was calculated as 6 years statistically, and mild group suffering from the disease for 6 years or less consisted of 17 patients (54.83%) with an age range of 45–78 years (mean age 63.72 ± 10.63) and the severe group with more than 6 years of the disease consisted of 14 patients (45.16%) with an age range of 47–83 years (mean age 69.80 ± 10.06, *p* = 0.104); mild tremor group (score 0-1-2) consisted of 21 patients (67.74%) with an age range of 45–79 years (mean age 65.68 ± 10.39) and the severe tremor group (score 3-4) consisted of 10 patients (32.25%) with an age range of 56–83 years (mean age 71.71 ± 9.77, *p* = 0.180); mild rigidity group (score 0-1-2) consisted of 20 patients (64.51%) with an age range of 45–79 years (mean age 65.25 ± 10.27) and the severe group consisted of 11 patients (35.48%) with an age range of 56–83 years (mean age 72.25 ± 9.55, *p* = 0.100). Each group was studied electrophysiologically.

In our study population, the statistical evaluation of the median of the duration of the disease as years was calculated as “6” years. The median and ulnar motor latencies at the wrist of the patients who had been suffering from the disease for more than 6 years were found to be significantly prolonged bilaterally.

According to the Hoehn and Yahr scores, bilateral median nerve motor latencies were found to be prolonged at the wrist significantly in the mild group (Table 5) suggesting a tendency to demyelination.

According to the rigidity and tremor scores, bilateral median nerve motor latencies at the wrist were found to be prolonged (Table 5) only in the mild group but not in the severe group.

There was no significant difference in ulnar nerve electrophysiologic parameters at the elbow level in the patient group neither regarding initial and latter affected sides nor regarding mild and severe groups and disease duration.

## 4. Discussion

In our study, we found the frequency of CTS as 16.12% is significantly prominent in older and more severe patients. A remarkable result was that CTS was bilateral among the two-thirds of the patients. As the prevalence of CTS in the general population has been reported to be 125 to 220 per 100000 [12], ratios mentioned above were significantly higher, calling attention to median neuropathy in PD. Similarly, Yucel et al. [4] found frequency of CTS as 24.4% electrophysiologically in their study where they reported a larger median nerve cross-sectional area sonographically in the severe PD patients. The anatomical site, wrist, was also evaluated in PD patients sonographically by Yang et al. [3] and median nerve cross-sectional area indicating median nerve swelling with edema was found to be larger (*p* < 0.05) in concordance with tremor severity due to the cumulative injury. In our study, although CTS was more prominent in patients suffering more severe disease, tremor and rigidity were not found to be contributing factors to median neuropathy evolution.

Rigidity, defined as a hypertonic state with increased resistance within the range of movement about a joint [13], may lead to tendon contractures and atrophy [14] and trigger the characteristic limb posture called striatal deformity accompanied by flexion at the elbow, ulnar deviation at the wrist, flexion at the metacarpophalangeal joints, and extension at the interphalangeal joints in varying severity [8]. Moreover, repetitive movement of the elbow superimposed by rest tremor at the wrist causes a narrowing of the tunnel and constriction of the nerve initiating a cycle of inflammation and oedema and increases the strain. The cubital tunnel cross-sectional area narrows by up to 55% as intraneural pressures increase up to 20-fold [15]. We confirmed ulnar neuropathy electrophysiologically as 3.22% in PD group whereas the incidence of the ulnar neuropathy (cubital tunnel syndrome) in the general population has been reported as 24.7 per 100.000 [16]. However, we did not find any association between neither rigidity and tremor severity nor H&Y scores and disease duration and ulnar neuropathy existence.

Our study's patient population (mean age 65.93 ± 10.58) and age-gender matched control group (mean age 62.03 ± 10.40, *p* = 0.145) consisted of elderly patients. We compared the mean indices of electrophysiologic parameters among each group for any subclinical involvement. Axonal degeneration causes decreased amplitude values whereas loose myelin causes prolonged distal motor latency and decreased sensorial nerve conduction velocities electrophysiologically in the peripheral nerves [17]. In regard to this, in the patient group, we evaluated significant median and ulnar demyelination at the wrist bilaterally and a tendency to ulnar nerve entrapment at the elbow being significant at the left and probable at the right. Advancing age factor in the development of entrapment neuropathy was excluded in the study population (*p* = 0.145). We call attention to the tendency of median and ulnar neuropathy in the PD group.

In our study, there was a relationship between median and ulnar nerve demyelination with both mild tremor and rigidity groups and mild H&Y group. As Parkinson's disease is progressive, mild symptomatology in the abovementioned groups confirms that these patients are at the early stages of the disease. Evidence suggests that peripheral nervous system involvement, sometimes occurring before clinical diagnosis, might be discussed as an intrinsic peripheral neuropathy before spreading of neurodegeneration to the central nervous system (CNS) [2]. Lewy body inclusions have been reported to be present both in the basal ganglia and in the peripheral nervous system [2]. Moreover, neuronal loss due to alpha-synuclein deposition, impaired axonal transport, and mitochondrial dysfunction causing neuronal death in the brain is being discussed as pathomechanisms of the neurodegenerative process in PD [2]. Doppler et al. [18] found alpha-synuclein deposition in cutaneous nerves of PD patients and speculated that both retrograde axonal degeneration due to impaired axonal transport and anterograde degeneration due to sensory neuronal cell death may lead to peripheral neuropathy and suggested that skin biopsy might be a useful diagnostic test for PD. Wang et al. [19] reported alpha-synuclein deposits in skin nerve fibers and suggested the possibility of a disease related peripheral neurodegeneration in PD.

Our results indicating no association with tremor and rigidity severity and mononeuropathy evolution are in accordance with the findings that in each individual PD patient suffering from different tremor and rigidity severity asymmetrically no difference was found in median and ulnar nerve neural transmission electrophysiologically. By this way, we excluded the extrinsic factors such as levodopa medication and intrinsic factors such as disease duration in each patient and had the opportunity to focus on symptom severity and asymmetry only.

In chronic demyelinating neuropathies, neuropathic tremor was reported in antagonist muscle pairs with a frequency of 3 to 6 Hz [20] which is similar to rest tremor. It has been proposed that distortion of the peripheral sensory input and feedback to the CNS, as the result of demyelination, leads to mismatch in spindle and tendon organ afferents and errors in the timing of muscle activity generated within the CNS [20, 21]. The tremor is most dominant in the upper extremities. As evaluated in our study, the demyelination of median and ulnar nerves especially at the wrist level might be an additive factor for tremor generation in the hands.

We observed median and ulnar demyelination at the wrist level in patients suffering from the disease for a longer period. The patients with a longer disease duration were not older than the ones with a shorter disease period (*p* = 0.104). In our study population, the mean of disease duration was calculated as 6 years statistically. In the literature, peripheral neuropathy with the advanced disease is mostly related with cumulative levodopa intake [2]. Chronic levodopa intake has been reported to increase homocysteine and reduce vit B12 serum levels suggesting a pharmacotoxic cause due to interactions of levodopa metabolism with the methylation pathways of vit B12. While polyneuropathy occurs mostly gradually and in subclinical form in patients on oral levodopa therapy, more severe acute/subacute forms were reported with enteral levodopa usage [2]. The question that is longer disease duration prone to polyneuropathy related to cumulative levodopa intake or is there a critic period when intrinsic factors contribute to polyneuropathy is open to discussion.

This study had some limitations. First, it will be necessary to validate these findings in a larger cohort. Second, as per this study, we aimed to evaluate if symptom severity has an additive effect on mononeuropathy generation; we did not focus on cumulative levodopa intake; and total time levodopa has been used.

In conclusion, this is the first study investigating symptom severity regarding tremor and rigidity as a potential risk for mononeuropathy generation in each individual patient. Although our results did not support the hypothesis that severity of rigidity and tremor might contribute to mononeuropathy evolution, we draw attention to disease related intrinsic factors in peripheral neurodegeneration. As PD has been proven to be a systemic disease not only related to CNS, future large-scale longitudinal studies focusing on pathophysiology of peripheral neurodegeneration in PD will provide a better understanding of the disease and will guide the clinician while applying patient specific therapeutic options in PD.

## Figures and Tables

**Table 1:** The clinical characteristics of the patients and the control group.

Variables	PD group (*n* = 31)	Control group (*n* = 32)	*p*
Gender (F/M)	18/13	22/10	
Age (year)	65.93 ± 10.58	62.03 ± 10.40	0.145
Age at onset (year)	59.48 ± 10.68		
PD duration (year)	6.45 ± 4.35		
Hoehn and Yahr stage	2.73 ± 0.94		
UPDRS part III score	21.86 ± 8.90		
Parkinson disease questionnaire	64.63 ± 24.09		

**Table 2:** Demographic and clinical characteristics of PD patients with median neuropathy versus without median neuropathy.

Variables	Median neuropathy (+)	Median neuropathy (−)	*p*
Mean age	77.00 ± 2.60	63.28 ± 10.03	**0.003**
PD duration (year)	8.50 ± 4.18	5.96 ± 4.33	0.205
Age at PD onset	68.50 ± 3.83	57.32 ± 10.70	**0.019**
Tremor score	1.83 ± 0.75	1.45 ± 0.97	0.390
Rigidity score	2.50 ± 0.54	1.79 ± 0.83	0.059
UPDRS part III score	30.66 ± 12.61	19.66 ± 6.33	**0.005**
Hoehn and Yahr stage	3.50 ± 1.04	2.54 ± 0.83	**0.023**
PDQ^a^	85.33 ± 15.43	59.45 ± 23.24	**0.016**

^a^Parkinson's disease questionnaire.

**Table 3:** The mean indices of electrophysiologic parameters between PD patients and controls.

	PD patients	Controls	*p*
Rmediandigit2velocity (s)	51.15 (41.10–60)	53.30 (40–64.90)	**0.049**
Lmediandigit2velocity (s)	51.10 (30–58.50)	52.20 (40–61.50)	**0.020**
Rulnardigit5wristvelocity (s)	53.70 (42.30–59.60)	55 (42.30–64.70)	0.250
Lulnardigit5wristvelocity (s)	53.03 ± 5.05	55.74 ± 4.93	**0.037**
RmedianAPBwristlatency (m)	3.63 ± 1.09	3.10 ± 0.42	**0.015**
RmedianAPBwristamp (m)	6.25 ± 2.53	7.52 ± 2.02	**0.033**
RmedianAPBelbowlatency (m)	7.36 ± 0.69	6.86 ± 0.81	**0.011**
RmedianAPBelbowampl (m)	5.68 ± 2.08	7.08 ± 2.06	**0.011**
RmedianAPBelbowvelocity (m)	57.52 ± 5.32	61.80 ± 8.19	**0.019**
LmedianAPBwristlatency (m)	3.35 (2.75–7.75)	3.00 (2.25–3.90)	**<0.001**
LmedianAPBwristampl (m)	6.37 ± 2.50	7.25 ± 2.16	0.145
LmedianAPBelbowlatency (m)	7.47 ± 1.16	6.76 ± 0.59	**0.004**
LmedianAPBelbowampl (m)	5.88 ± 2.21	6.64 ± 2.06	0.228
LmedianAPBelbowvelocity (m)	57.80 (44–184)	62.90 (51.70–73.30)	0.091
RulnarADMwristlatency (m)	2.55 (2.05–3.20)	2.35 (1.85–3.50)	**0.023**
RulnarADMwristampl (m)	9.31 ± 1.39	9.66 ± 1.52	0.351
RulnarADMb.elbowvelocity (m)	60.30 (50.50–78.80)	62 (51.10–94.10)	0.176
RulnarADMa.elbowvelocity (m)	58.50 ± 1.28	58.77 ± 1.60	0.148
LulnarADMwristlatency (m)	2.60 (2.15–4)	2.35 (2–3.30)	**0.011**
LulnarADMwristampl (m)	10.10 (6.10–89)	10 (6.40–14.30)	0.607
LulnarADMb.elbowvelocity (m)	58.30 (41.20–77.20)	63 (55.80–83)	**0.002**
LulnarADMa.elbowvelocity (m)	53.80 (35.90–73.70)	57.10 (41.90–80)	**0.017**

(s): sensorial; (m): motor; R: right; L: left; ampl: amplitude; APB: abductor pollicis brevis; ADM: adductor digiti minimi; a: after; b: below.

**Table 4:** Correspondence of mean indices of electrophysiologic parameters between initial and later symptomatic sides in PD patients.

	Initial symptomatic side	Later symptomatic side	*p*
mediandigit2latency (s)	2.76 ± 0.08	2.53 ± 0.42	0.523
mediandigit2velocity (s)	51.41 ± 3.89	49.50 ± 6.15	0.082
mediandigit2peakampl (s)	37.36 ± 16.38	35.16 ± 18.86	0.457
ulnardigit5wristlatency (s)	2.11 ± 0.23	2.13 ± 0.23	0.528
ulnardigit5wristvelocity (s)	53.55 ± 4.58	52.83 ± 4.65	0.295
ulnardigit5wristpeakampl (s)	29.18 ± 14.93	29.83 ± 14.40	0.777
ulnarlatency (m)	2.22 ± 0.32	2.22 ± 0.21	0.971
ulnarpeakampl (m)	23.79 ± 15.84	20.20 ± 14.28	0.052
medianlatency (m)	2.54 ± 0.29	2.53 ± 0.26	0.927
medianpeakampl (m)	20.11 ± 12.30	18.26 ± 11.70	0.289
medianAPBwristlatency (m)	3.63 ± 1.12	3.73 ± 1.11	0.475
medianAPBwristamp (m)	6.61 ± 2.52	6.00 ± 2.51	0.257
medianAPBelbowlatency (m)	7.32 ± 0.70	7.53 ± 1.17	0.636
medianAPBelbowampl (m)	6.05 ± 2.04	5.49 ± 2.24	0.406
medianAPBelbowvelocity (m)	57.66 ± 5.21	62.24 ± 23.79	0.252
ulnarADMwristlatency (m)	2.62 ± 0.32	2.61 ± 0.41	0.884
ulnarADMwristampl (m)	9.44 ± 1.73	12.44 ± 14.58	0.288
ulnarADMb.elbowlatency (m)	6.41 ± 0.65	6.42 ± 0.83	0.83
ulnarADMb.elbowampl (m)	8.83 ± 1.58	8.88 ± 1.88	0.882
ulnarADMb.elbowvelocity (m)	59.81 ± 6.51	59.46 ± 7.65	0.756
ulnarADMa.elbowlatency (m)	7.85 ± 0.80	7.88 ± 1	0.823
ulnarADMa.elbowampl (m)	8.52 ± 1.53	8.51 ± 1.84	0.982
ulnarADMa.elbowvelocity (m)	54.85 ± 5.97	55.24 ± 7.15	0.79

(s): sensorial; (m): motor; ampl: amplitude; APB: abductor pollicis brevis; ADM: adductor digiti minimi; b: before; a: after.

**Table 5:** The statistical comparison of mean indices of median and ulnar nerve electrophysiologic parameters at the wrist level between mild and severe patient groups and controls.

Variables	Disease duration (years)		Hoehn & Yahr score		Rigidity		Tremor	
	Shorter than 6 years	Longer than 6 years	Mild	Severe	Mild	Severe	Mild	Severe
m-dml								
R	NS	0.002	0.041	NS	0.007	NS	0.003	0.003
L	NS	0.001	0.002	0.002	0.001	0.001	0.002	NS
m-amp								
R	NS	NS	NS	0.041	NS	NS	NS	NS
L	NS	NS	NS	NS	NS	NS	NS	NS
m-ncv								
R	NS	NS	NS	0.035	NS	NS	NS	NS
L	NS	NS	NS	NS	NS	NS	NS	NS
u-dml								
R	NS	0.037	NS	NS	NS	0.049	NS	NS
L	NS	0.008	NS	0.025	0.034	NS	NS	0.011
u-amp								
R	0.031	0.031	NS	NS	NS	NS	NS	NS
L	NS	NS	NS	NS	NS	NS	NS	NS
u-ncv								
R	NS	NS	NS	NS	NS	NS	NS	NS
L	NS	NS	NS	NS	NS	NS	NS	NS

m: median; dml: distal motor latency; amp: amplitude; ncv: nerve conduction velocity; u: ulnar; NS: not significant; R: right; L: left.
